# Long-term Health Outcomes Among Survivors Exposed to Sulfur Mustard in Iran

**DOI:** 10.1001/jamanetworkopen.2020.28894

**Published:** 2020-12-10

**Authors:** Hossein Amini, Masoud Solaymani-Dodaran, Batool Mousavi, Seyed Nooredin Alam Beladi, Mohammad Reza Soroush, Jamileh Abolghasemi, Amir Vahedian-Azimi, Mahmoud Salesi, Paul C. Guest, Amirhossein Sahebkar, Mostafa Ghanei

**Affiliations:** 1 Department of Epidemiology, School of Public Health, Iran University of Medical Sciences, Tehran, Iran; 2 Minimally Invasive Surgery Research Center, Rasoul Hospital, Iran University of Medical Sciences, Tehran, Iran; 3 Division of Epidemiology and Public Health, the University of Nottingham, United Kingdom; 4 Prevention Department, Janbazan Medical and Engineering Research Center, Tehran, Iran; 5 Department of Biostatistics, School of Public Health, Iran University of Medical Sciences, Tehran, Iran; 6 Trauma Research Center, Nursing Faculty, Baqiyatallah University of Medical Sciences, Tehran, Iran; 7 Chemical Injuries Research Center, Systems Biology and Poisonings Institute, Baqiyatallah University of Medical Sciences, Tehran, Iran; 8 Department of Biochemistry and Tissue Biology, Institute of Biology, University of Campinas, Campinas, São Paulo, Brazil; 9 Biotechnology Research Center, Pharmaceutical Technology Institute, Mashhad University of Medical Sciences, Mashhad, Iran; 10 Neurogenic Inflammation Research Center, Mashhad University of Medical Sciences, Mashhad, Iran; 11 Polish Mother’s Memorial Hospital Research Institute, Lodz, Poland

## Abstract

**Importance:**

The prevalence and severity of long-term health complications after exposure to sulfur mustard are unknown.

**Objective:**

To investigate the long-term health outcomes among survivors exposed to sulfur mustard during the Iran-Iraq War.

**Design, Setting, and Participants:**

In this retrospective cohort study, late-onset health complications of 64 190 Iranian survivors exposed to sulfur mustard during the Iran-Iraq War from 1980 to 1988 were investigated using descriptive statistics. Data involving affected organs and symptom severity were extracted from the Veterans and Martyr Affair Foundation (VMAF) database from 1980 to 2019. Assessments were conducted across 3 groups depending on whether survivors were (1) evacuated and admitted (EA) to a hospital; (2) not evacuated or admitted (NEA) to a hospital; or (3) evacuation or admission status was not documented.

**Exposures:**

Analysis of chronic symptom severity following exposure to sulfur mustard.

**Main Outcomes and Measures:**

Mild, moderate, or severe rankings of symptoms in lungs, eyes, and skin of survivors exposed to sulfur mustard using data from the VMAF database.

**Results:**

Of 64 190 chemical survivors registered in the VMAF database, 60 861 met the inclusion criteria. Of the included survivors, 98.0% were male, and the mean (SD) age was 23.5 (7.7) years. Most survivors (53 675 [88.2%]) had no symptoms or mild lesions, and 7186 survivors (11.8%) had moderate or severe complications. Moderate to severe lung (6540 [10.7%]), eye (335 [0.6%]), or skin (725 [1.2%]) injuries were documented in the exposed population. The proportion of moderate plus severe late complications in eyes was 3 times as high in male survivors compared with female survivors (0.6% [95% CI, 0.53%-0.65%] vs 0.2% [95% CI, 0.09%-0.73%]; *P* < .001), whereas dermal complications were significantly more common in female survivors (3.9% [95% CI, 2.92%-5.11%] vs 1.14% [95% CI, 1.06%-1.23%]; *P* < .001). Mild lung lesions were more prevalent in the NEA group than in the EA group (73.9% [95% CI, 73.4%-74.4%] vs 11.0% [95% CI, 10.6%-11.3%]; *P* < .001). In the NEA group, 83.2% (n = 23 866) developed lung injuries that were mostly mild or moderate, whereas 77% (n = 24 766) of the EA group did not develop lung injuries (*P* < .001).

**Conclusions and Relevance:**

The present study found sex differences in the frequencies of eye and skin complications following sulfur mustard exposure, and lung complications were more prevalent years after sulfur mustard exposure than soon after exposure. Mild lung lesions were observed more frequently among sulfur mustard–exposed survivors who had not been evacuated or hospitalized than among those who had been evacuated or hospitalized. These differences may be due to physiological response or dose of exposure. Close monitoring over an extended period may be required for detection of late pulmonary complications in individuals exposed to sulfur mustard.

## Key Points

**Question:**

What are the long-term health complications associated with sulfur mustard exposure, and are there differences associated with exposure levels or demographic characteristics?

**Findings:**

In this cohort study of 64 190 chemical warfare survivors of the Iran-Iraq War from 1980 to 1988 registered in the Veterans and Martyr Affair Foundation database, there were sex differences in the frequencies of eye and skin complications among survivors of sulfur mustard exposure. Lung complications were more apparent in later years following sulfur mustard exposure in survivors who had no initial signs of distress, and these complications were observed more among persons who were not evacuated or hospitalized than among those who were.

**Meaning:**

Differences in health complications may be due to distinct physiological responses or levels of chemical exposure, and close monitoring even decades after sulfur mustard exposure is required to detect late pulmonary complications.

## Teaser

This cohort study investigates the prevalence by sex, age, and symptom severity of long-term lung, eye, and skin complications among survivors exposed to sulfur mustard between 1980 and 1988 during the Iran-Iraq War.

## Introduction

Chemical weapons are among the most inhumane forms of warfare owing to their severe acute and chronic complications in survivors of these attacks.^1,2^ The most well-known chemical weapons include nerve agents and sulfur mustard (SM).^3^ The first use of such weapons after World War II dates back to the Iran-Iraq War in the 1980s. The years 1984, 1986, and 1987 were the pinnacle of the use of such weapons as confirmed by United Nations experts through field investigations, clinical examination of the exposed persons, and examination of Iraq’s munitions and ammunitions.^4^

In the Iran-Iraq War, the Iraqi regime used SM from 1980 to 1988 in different military operations.^5,6^ This gas is a strong alkalinizing agent, which results in cellular necrosis through its linking to cellular protein components and nucleic acids, thereby causing complications that can induce mutagenic and carcinogenic consequences.^7,8,9^ It was synthesized first by Despretz in 1822^10^ and used by the German army for the first time in 1917 against Canadian soldiers and then French soldiers. The British army used it in 1918 in the Hindenburg War. From then on, countries including Spain (1923-1926), Italy (1935-1940), the Soviet Union (1930), Japan (1937-1945), and Egypt (1963-1967) have used this chemical agent in warfare,^11^ leaving more than 400 000 individuals exposed so far.^12^ Given the effects on exposed humans, many national and international efforts have been made or attempted to prevent the use of such weapons in military conflicts.^13,14^

The research carried out so far indicates that SM would produce different effects of varying severity in 3 vital organs: the lungs, eyes, and skin. The severity and rate of damage in each survivor exposed to SM depends on various factors, such as the quantity, length, and route of exposure to the agent; exposure to other chemical agents; age and sex; and nutritional and health status. Some of these factors have been correlated with the frequency of injuries, primary symptoms, consumption of drugs, and length of hospital stay.^15,16^ The acute local effects of exposure are due to contact with relatively high concentrations of the agent, manifesting mostly as dermal, ocular, and respiratory lesions. These lesions can increase the chances of death when they are sufficiently severe. The chronic consequences of SM may be observed as complications even up to 50 years after exposure. These complications mostly affect the respiratory system and can include chronic bronchitis, bronchiectasis, frequent bronchopneumonia, and pulmonary fibrosis, all of which become worse over time.^1,2,17^

Although there are numerous reports on the short- and long-term complications in various organ systems among survivors of SM exposure during the Iran-Iraq War,^1,5,6,18,19,20,21,22^ the prevalence and severity of these complications are unknown. Here, we carried out a retrospective cohort study of 64 190 chemical warfare survivors of this war using the Veterans and Martyr Affair Foundation (VMAF) database. The primary objectives were to investigate the frequencies of acute and long-term complications among individuals exposed to SM based on age, sex, severity of injury, organ injured, geographic region of SM exposure, and documented SM exposure confirmation.

## Methods

### Study Design and Setting

A retrospective longitudinal cohort study was conducted with survivors who were exposed to chemical warfare agents during the Iran-Iraq War from 1980 to 1988. All data of survivors exposed to chemical warfare agents were included apart from individuals exposed to nerve agents, and all data are registered in the VMAF. Some survivors had medical documents related to the time of exposure, when they were first hospitalized in the field or in an urban hospital because of ocular, pulmonary, or cutaneous complications.

Injured survivors registered with the VMAF to receive free health care services. This agency serves war survivors and their families by consistently providing the highest quality of long-term care services.^7^ The primary foundation duties include securing pensions, establishing a research center, and providing health insurance coverage, rehabilitation services, a medical commission consisting of physicians and specialists to periodically examine and record related complications, habitation, employment support, social-cultural and sports services, and nursing home services.

Evidence of late-onset complications was examined from 1980 through March 31, 2019 (39 years). This study followed the Strengthening the Reporting of Observational Studies in Epidemiology for Respondent-Driven Sampling Studies (STROBE-RDS) statement reporting guideline.^23^ The study was approved by the institutional review board at Iran University of Medical Sciences and Janbazan Medical and Engineering Research Center, Tehran, Iran, which waived the need for obtaining informed consent because this study used nonidentifiable data from an existing data set.

### Verification of SM Exposure

Based on the evidence of exposure to SM, the data were categorized into 3 groups: (1) evacuated and admitted (EA), confirmed history of exposure in the affected geographic region with evacuation and hospital admission; (2) not evacuated or admitted (NEA), confirmed history of exposure without evacuation or hospital admission; and (3) undocumented cases, not classified in the other 2 groups but showing late clinical manifestations suggestive of exposure.

### Verification of SM Injuries

All war-associated injuries were recorded in the VMAF database. The diagnosis of injuries and severity of lesions in the lungs, skin, and eyes were determined by a panel of medical experts (which included M.G.).

Classification criteria for chemical warfare survivors were similar to proposed criteria by the Global Initiative for Chronic Obstructive Lung Disease, which categorizes patients with chronic obstructive pulmonary disease into 4 groups based on signs and symptoms.^24,25^ The members of the expert panel included a pulmonologist (M.G.), psychiatrist, neurologist, ophthalmologist, dermatologist, cardiologist, forensic medicine specialist, and nephrologist. The expert panel investigated the documents case by case and recorded complications associated with chemical injuries in an ongoing manner. The symptomatic nature of the acute and long-term complications of SM exposure prompts exposed individuals to be referred to the VMAF to obtain supportive care. Therefore, it is likely that the overall number of exposed individuals does not differ much from the number of registered individuals in the database.

### Definition of SM Organ Injuries

#### Lungs

The specific clinical sign of SM in the lungs was presence of obliterative bronchiolitis syndrome on high-resolution computed tomography (HRCT) analysis of the lungs in the absence of confounding factors, such as smoking, occupational hazards, or underlying diseases.^26^ Lung lesions following SM exposure were categorized as determined by spirometry results of forced expiratory volume in the first second (FEV1) as follows: no symptoms, FEV1 higher than 80%; mild, FEV1 higher than 70% but lower than or equal to 80%; moderate, FEV1 higher than 50% but lower than or equal to 70%; and severe, FEV1 lower than or equal to 50% with normal arterial blood gas or severe tracheobronchomalacia.

#### Skin

The presence of SM-specific scars (pigmentation, vascular, or trophic changes) in areas of the skin where they would commonly be observed or the appearance of cutaneous T-cell lymphoma or mycosis fungoides represent the defining clinical signs and symptoms of SM exposure on the skin.^27^ The mild category was defined as the presence of an SM-specific scar across less than 5% of the body surface; the presence of such a scar plus mild dermatitis and mild xerosis or mild-to-moderate autoimmune diseases; and concurrent medical documents plus severe autoimmune diseases or any mild dermatitis. The moderate category was defined as the presence of an SM-specific scar affecting between 5% and 20% of the body surface^2^; presence of an SM-specific scar with significant trophic changes in the genital or anus areas; the presence of an SM-specific scar of any size with or without concurrent medical documents plus severe dermatitis covering less than 50% of the body surface; concurrent medical documents with severe dermatitis or xerosis covering less than 50% of the body surface; and the presence of an SM-specific scar of any size with or without concurrent medical documents plus any severe autoimmune disease affecting the skin covering more than 50% of the body surface. The severe category was defined as the presence of an SM-specific scar covering more than 20% of the body surface; the presence of an SM-specific scar of any size with or without concurrent medical documents with severe dermatitis or xerosis covering more than 50% of the body surface; and concurrent medical documents with severe dermatitis or xerosis covering less than 50% of the body surface.

#### Eyes

The specific clinical signs and symptoms of SM in the eyes include the presence of conjunctiva vascular changes (telangiectasia, segmentation, venous beading, tortuosity, and vascular dilatation) along with the vascular ischemic complications of limbus.^28^ The mild category was defined as burning, itching, tearing, redness, foreign body sensation, blurred vision, conjunctivitis, subconjunctival hemorrhage, photophobia, or conjunctival vascular changes (telangiectasia and vascular changes, edema of the eyelid, papillary changes) of 1 or both eyes. The moderate category was defined as the same changes in the mild category along with conjunctival ischemia in the limbus area, as well as unilateral or bilateral closure or cauterization of punctum. The severe category was defined as those observations in the moderate category along with symptoms of corneal involvement, epithelial and subepithelial opacity and anterior stroma in the cornea, keratopathy, pannus, hyperpigmentation around the nerve, iron deposition in the cornea and corneal vascularization, stenosis, and involvement of less than half unilaterally or bilaterally in both eyes.

### Statistical Analysis

All analyses were performed using SPSS, version 23.0 (IBM Corp), and descriptive statistics were calculated for all variables, including absolute numbers and percentages. The frequencies of chemical injuries among the study population were determined according to age, sex, and organ, as well as by severity of injury. In addition, the characteristics of the study population were determined according to injured organ and documented exposure level. A 2-tailed *P* < .05 was considered statistically significant.

## Results

In total, 64 190 chemical warfare survivors were analyzed, of whom 60 861 were included in the final analyses. According to verified information from the VMAF database, there were 2205 survivors who had been exposed to nerve agents, and 1124 individuals were undocumented. There was a male predominance (59 648 [98.0%]), and the mean (SD) age at the time of exposure was 23.5 (7.7) years. The frequencies of severity of the SM injuries by age and by sex at the time of exposure are presented in Table 1. The median (interquartile range) age at the time of exposure was 21 (19-26) years.

**Table 1.** Distributions of Sulfur Mustard Injuries Among Study Population by Age at Time of Exposure and Sex

Lesion category	No. (%) of exposed survivors						
	Male age, y			Female age, y			Total
	≤21	>21	All	≤21	>21	All	
None	9726 (38.6)	8075 (37.2)	17 801 (38.0)	134 (32.1)	142 (27.2)	276 (29.4)	18 077 (37.8)
Mild	12 761 (50.6)	10 626 (49.0)	23 387 (49.9)	225 (54.0)	310 (59.3)	535 (56.9)	23 922 (50.0)
Moderate	2435 (9.7)	2691 (12.4)	5126 (10.9)	52 (12.5)	66 (12.6)	118 (12.6)	5244 (11.0)
Severe	274 (1.1)	301 (1.4)	575 (1.2)	6 (1.4)	5 (1.0)	11 (1.2)	586 (1.2)
Total	25 196 (100)	21 693 (100)	46 889 (100)	417 (100)	523 (100)	940 (100)	47 829 (100)
*P* value for χ^2^ for trend	<.001			<.001			

The overall numbers of individuals and the mean (SD) ages in each symptom group were as follows: no symptoms, 18 077 individuals aged 23.37 (7.55) years; mild, 23 922 individuals aged 23.22 (7.36) years; moderate, 5244 individuals aged 24.86 (9.00) years; and severe, 586 individuals aged 24.75 (8.80) years. Overall, of the 60 861 studied chemical survivors, 53 675 were categorized in the groups of no symptoms (40.1%) or mild symptoms (48.1%), and 7186 were placed in the groups of moderate or severe symptoms (11.8%). For lung injuries, 29 587 individuals (48.6%) showed no symptoms, whereas 24 734 individuals (40.6%) had mild symptoms, 6036 individuals (9.9%) had moderate symptoms, and 504 individuals (0.8%) had severe symptoms. For eye injuries, 52 632 individuals (86.5%) had no symptoms, 7874 individuals (12.9%) had mild injuries, 175 individuals (0.3%) had moderate injuries, and 180 individuals (0.3%) had severe injuries. For skin injuries, 54 319 individuals (89.3%) had no symptoms, whereas 58.17 individuals (9.6%) had mild lesions, 621 individuals (1.0%) had moderate lesions, and 104 individuals (0.2%) had severe lesions.

The frequencies of lesions in the lungs, eyes, and skin according to age and sex are presented in Table 2. The proportion of moderate and severe late complications in eyes was 3 times as high in male vs female survivors (0.6% [95% CI, 0.53%-0.65%] vs 0.2% [95% CI, 0.09%-0.73%]; *P* < .001). By contrast, dermal complications were 3 times as high in female vs male survivors (3.9% [95% CI, 2.92%-5.11%] vs 1.14% [95% CI, 1.06%-1.23%]; *P* < .001) (Table 2). The distributions of no symptoms to severe symptoms are shown in Table 3 in the 2 exposure groups for lung, eye, and skin injuries. Mild lung lesions were more prevalent in the NEA group compared with the EA group (73.9% [95% CI, 73.4%-74.4%] vs 11.0% [95% CI, 10.6%-11.3%]; *P* < .001). In the NEA group, 83.2% (n = 23 866) developed lung injuries that were mostly mild or moderate, whereas 77% (n = 24 766) of the EA group did not develop lung injuries (*P* < .001). The proportions of moderate and severe late complications in eyes, lungs, and skin across 31 Iranian provinces are displayed in the Figure. The highest proportions of SM injuries were reported in West Azerbaijan (16.5%), Chahar Mahal and Bakhtiyari (16.1%), Kermanshah (15.8%), Ilam (15.8%), East Azerbaijan (14.5%), Kurdistan (14.4%), Yazd, (13.8%), and Sistan and Baluchistan (13.1%) provinces. Furthermore, the highest numbers of Iranian SM warfare survivors were found in Isfahan (n = 6248), Tehran (n = 5218), Khorasan Razavi (n = 4397), Mazandaran (n = 4372), and Kerman (n = 4179).

**Table 2.** Distribution of Lesions in Injured Organs According to Age, Sex, and Injury Severity

Symptom category	Eye, No. (%) of exposed survivors				Skin, No. (%) of exposed survivors					Lung, No. (%) of exposed survivors				
	Age, y		Sex		Age, y		Sex		Age, y			Sex	
	≤21	>21	Male	Female	≤21	>21	Male	Female	≤21		>21	Male	Female
None	21 816 (85.2)	19 022 (85.6)	51 546 (86.4)	1086 (89.5)	22 950 (89.6)	19 728 (88.8)	53 344 (89.4)	975 (80.4)	12 131 (47.4)		10 159 (45.7)	29 138 (48.8)	449 (37.0)
Mild	3635 (14.2)	3059 (13.8)	7750 (13.0)	124 (10.2)	2380 (9.3)	2190 (9.9)	5626 (9.4)	191 (15.7)	10 977 (42.9)		9246 (41.6)	24 110 (40.4)	624 (51.4)
Moderate	63 (0.2)	74 (0.3)	172 (0.3)	3 (0.2)	242 (0.9)	252 (1.1)	581 (1.0)	40 (3.3)	2341 (9.1)		2599 (11.7)	5906 (9.9)	130 (10.7)
Severe	99 (0.4)	61 (0.3)	180 (0.3)	0	41 (0.2)	46 (0.2)	97 (0.2)	7 (0.6)	164 (0.6)		212 (1.0)	494 (0.8)	10 (0.8)
Total	25 613 (100)	22 216 (100)	59 648 (100)	1213 (100)	25 613 (100)	22 216 (100)	59 648 (100)	1213 (100)	25 613 (100)		22 216 (100)	59 648 (100)	1213 (100)
*P* value for χ^2^ for trend	<.001		<.001		<.001		<.001		<.001			<.001	

**Table 3.** Characteristics of Study Population According to Injured Organ and Documented Exposure Level

Characteristic	No. (%) of exposed survivors		
	Evacuated and admitted	Not evacuated or admitted	Total
Age group, y			
≤21	13 540 (53.8)	12 073 (53.2)	25 155 (52.6)
>21	11 615(46.2)	10 601 (46.8)	22 674 (47.4)
Total	25 155 (100)	22 674 (100)	47 829 (100)
Sex			
Male	31 781 (98.7)	27 867 (97.1)	59 648 (98)
Female	393 (1.3)	820 (2.9)	1213 (2)
Total	32 174 (100)	28 687 (100)	60 861(100)
Lesion			
Late lung			
None	24 766 (77)	4821 (16.8)	29 587 (48.6)
Mild	3536 (11)	21 198 (73.9)	24 734 (40.6)
Moderate	3378 (10.5)	2658 (9.3)	6036 (9.9)
Severe	494 (1.5)	10 (0)	504 (0.8)
Late skin			
None	27 164 (84.4)	27 155 (94.7)	54 319 (89.3)
Mild	4435 (13.8)	1382 (4.8)	5817 (9.6)
Moderate	484 (1.5)	137 (0.5)	621 (1)
Severe	91 (0.3)	13 (0)	104 (0.2)
Late eye			
None	25 194 (78.3)	27 438 (95.6)	52 632 (86.5)
Mild	6683 (20.8)	1191 (4.2)	7874 (12.9)
Moderate	135 (0.4)	40 (0.1)	175 (0.3)
Severe	162 (0.5)	18 (0.1)	180 (0.3)
*P* value for χ^2^ for trend	<.001	<.001	

**Figure.**
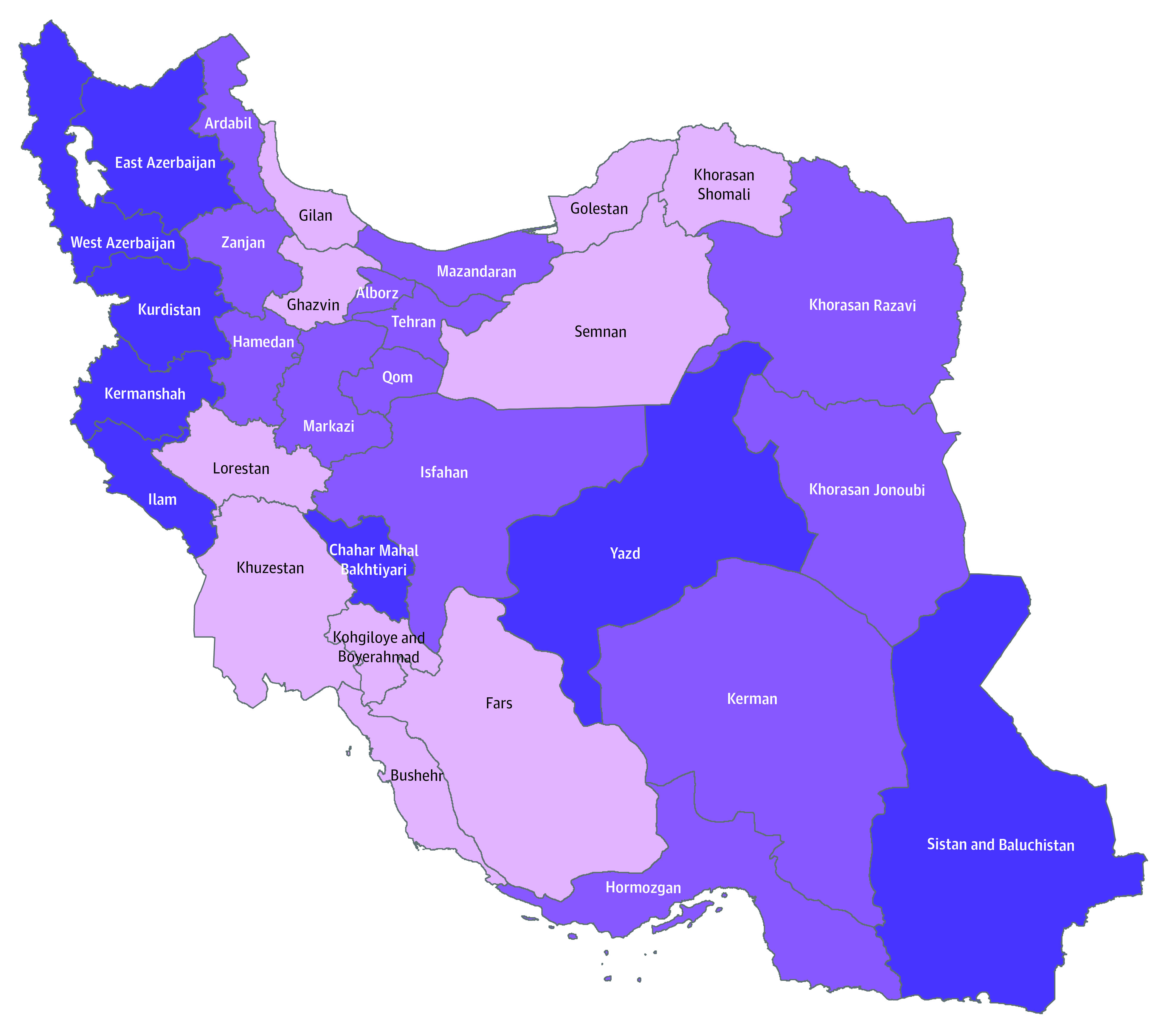
Proportion of Lung, Eye, and Skin Moderate and Severe Late Complications of Sulfur Mustard in 31 Iranian Provinces Light purple indicates less than 10%; purple, between 10% and 13%; and dark purple, more than 13%.

## Discussion

The present study found that of 60 861 survivors of SM exposure, most had no symptoms (40.1%) or mild symptoms (48.1%), with 11.8% experiencing moderate to severe symptoms. We believe that the provision of immediate and effective care under the national care management scheme contributed substantially to the high proportion of survivors with mild or no symptoms. However, exposure to chemicals is the main reason that chemical warfare survivors need frequent health care, thereby imposing high costs on the health care system.^1^

The present study showed that the severity of the exposure was associated with the type and severity of lesion. As expected, the proportions of moderate and severe complications in all 3 organs were higher in the EA group. Mild lung lesions were more prevalent in the NEA group than in the EA group (73.9% vs 11.0%). Thus, mild pulmonary complications became apparent in the years following SM exposure in numerous individuals who did not at first experience signs of distress. Furthermore, the data indicated that persons with eye and skin lesions were referred to a health center sooner than individuals with pulmonary injury, which manifested itself years later. Thus, overall, these data suggest that in a chemical attack, a healthy exposed population will likely develop no or mild pulmonary complications.

Sex was associated with the type and severity of lesion. Considering moderate to severe complications, the present study found that men had approximately 3 times as many eye complications as women. By contrast, dermal complications were approximately 3 times as high in women compared with men (Table 2). There has been a lack of similar studies addressing sex differences in long-term effects and complications after exposure to SM. These differences may be due to differing physiological responses, dose of exposure, or other factors that warrant further studies.

Many environmental factors, such as air pollution, temperature, light, wind, and humidity, can influence the symptoms of exposure to SM.^14^ In addition, the severity of these lesions and the rate of damage sustained by each survivor exposed to this agent depend on factors that include the amount of agent used, length of exposure, route of contact, exposure to other chemicals, age, sex, nutrition, lifestyle, and health status of the individual. All these factors have been associated with the injury frequency rate, primary symptoms, drug consumption, and length of hospital stay.^15,16^ In addition, the higher percentages of injuries reported in Iranian provinces that share a border with Iraq are likely associated with a greater chance of exposure to chemical attacks in those regions.

The frequencies of chemical injuries of various body organs have been reported in several studies. The highest proportion corresponded to pulmonary injuries (86.4%), followed by dermal and ocular symptoms.^29^ The most common pulmonary complications associated with SM exposure are biochemical disturbances of the lungs, pulmonary vascular damage, and impaired immune system of the lungs.^1,15,19,20^ In the lungs, SM has been associated with severe inflammation of the tracheobronchial epithelium with heavy leukocyte infiltration, alveolar hemorrhage with thrombi formation, and vacuolation of lung parenchymal cells.^30^ Chronic bronchitis is reported to be the most common chronic pulmonary complication associated with SM exposure, with a frequency of 50%.^31^ It is believed that this disorder is often associated with considerable physical disability.^12^ Bronchial asthma, hypersensitivity to inhalational stimulators, and increased risk of respiratory infections have been reported in this population.^12^ The pulmonary complications of SM exposure are typically associated with the concentration and the duration of exposure.^32^

The second late complication of chemical injury occurs in the skin system.^1,17,19,20^ Severe inflammation of the skin associated with SE exposure is caused by induction of the proinflammatory cytokines interleukins 1β, 6, and 8 and tumor necrosis factor α.^33^ A study by Vogt et al^34^ examined skin lesions induced by SM and found that the dermal reaction to SM poisoning has 2 phases. The primary or immediate phase is characterized by damage to fibroblasts as well as to endothelium of superficial capillaries and venules, which is due to cell membrane damage. The second or late phase is characterized by death of the epidermal basal cells due to DNA damage, vascular infiltrate, migration of neutrophils, abnormal activity of fibroblasts, and finally ulcer and scar formation.

The ocular complications of SM were investigated for the first time in World War I.^32^ Ocular alterations can manifest themselves even 50 years after exposure.^34^ Although ocular tissue is highly sensitive to SM, which can cause severe conjunctival and scleral pain, inflammation, lacrimation, blepharospasm, and photophobia, these effects may be evident for no longer than 1 hour.^35^ Sulfur mustard attaches to corneal collagens, where the sulfur-containing mustard collagen induces physical and chemical changes that cause continual irritation of the eyes, reduced corneal sensation, vascular changes, impaired regional blood circulation, creation and healing of wounds, recurrent attacks, thinning and eventual perforation of the cornea, and degradation of the cornea.^32,36^

### Limitations and Strengths

The limitations^1^ of the study included the lack of information on the following variables: the course of progression and incidence of late complications,^2^ the rate of immediate complications at the time of exposure,^3^ the dose of SM at exposure,^4^ the time interval until evacuated and admitted to a hospital,^5^ the proximity to the attacked zone,^6^ and the use of protective equipment such as masks.^7^ Another limitation was the failure to acquire access to data on all survivors exposed to SM despite the use of formal data available in the VMAF.

The strengths of the study include the large study size (64 190 chemical warfare survivors). The severities of complications in the population were assessed based on expert diagnosis of the VMAF Medical Commission using guidelines for determining the rates of chemical damage. The present study included the entire population of chemical warfare survivors in Iran registered in the VMAF, the only official source for providing information regarding this topic. Therefore, the studied population represents the total number of documented exposed individuals. Diagnosis of the injuries and severity of the lesions in the lungs, skin, and eyes were determined by a panel of medical experts. The database included information that covered both military and civilian male and female individuals who were exposed to SM during the Iran-Iraq War.

## Conclusions

The present study was the first to our knowledge to investigate chemical warfare survivors and classify injury severity according to their documented exposure level and sex and to identify the frequencies of complications in 31 provinces of Iran. Survivors exposed to SM during the Iran-Iraq War were classified into 1 of 2 groups with respect to exposure, enabling more accurate assessment of injuries following the exposure in different subgroups of the population. Most of the survivors had mild or no symptoms, whereas 12% had moderate or severe symptoms. The present research will inform the creation of an applied database of chemical warfare survivors and the design of therapeutic interventions and periodic examinations to reduce the late pulmonary, dermal, and ocular complications in different subgroups of the exposed population.
